# Author Correction: Turning up the heat mimics allosteric signaling in imidazole-glycerol phosphate synthase

**DOI:** 10.1038/s41467-023-38370-3

**Published:** 2023-05-05

**Authors:** Federica Maschietto, Uriel N. Morzan, Florentina Tofoleanu, Aria Gheeraert, Apala Chaudhuri, Gregory W. Kyro, Peter Nekrasov, Bernard Brooks, J. Patrick Loria, Ivan Rivalta, Victor S. Batista

**Affiliations:** 1 Department of Chemistry, Yale University, P.O. Box 208107, New Haven, CT 06520-8107 USA; 2 International Center for Theoretical Physics, Strada Costiera 11, 34151 Trieste, Italy; 3 Laboratory of Computational Biology, National Heart, Lung and Blood Institute, National Institutes of Health, Bethesda, MD 20852 USA; 4 ENSL, CNRS, Laboratoire de Chimie UMR 5182, 46 allée d’Italie, 69364 Lyon, France; 5 Dipartimento di Chimica Industriale “Toso Montanari”, Alma Mater Studiorum, Università di Bologna, Bologna, Italy; 6 Department of Molecular Biophysics and Biochemistry, Yale University, New Haven, CT 06520 USA; 7 Present Address: Treeline Biosciences, 500 Arsenal Street, Watertown, MA 02472 USA

**Keywords:** Computational biophysics, Molecular conformation, Biophysical chemistry, Solution-state NMR

Correction to: *Nature Communications* 10.1038/s41467-023-37956-1, published online 19 April 2023

The original version of this Article contained an error in Fig. 4a, in which the colors in the plot were not defined.

This has been corrected in both the PDF and HTML versions of the Article.

