# Supplementary material for: Turning up the heat mimics allosteric signaling in imidazole-glycerol phosphate synthase
Source: Nat Commun. 2023 Apr 19;14:2239. doi: 10.1038/s41467-023-37956-1 (PMC10115891; doi:10.1038/s41467-023-37956-1)
Supplement: Supplementary file 1 — Supplementary Information [file 41467_2023_37956_MOESM1_ESM.pdf]

Supporting Information for: Turning Up the Heat Mimics Allosteric Signaling in Imidazole-Glycerol Phosphate Synthase

Federica Maschietto,[*,1,a] Uriel N. Morzan,[*,2,a] Florentina Tofoleanu,[*,1,3,] ✝ Aria Gheeraert,[*,4,5] Apala Chaudhuri,[*,6] Gregory W. Kyro,[1] Peter Nekrasov,[1] Bernard Brooks,[1] J. Patrick Loria,[1,6,a] Ivan Rivalta,[5,a] Victor S. Batista[1,a]

1. Department of Chemistry, Yale University, P.O. Box 208107, New Haven, CT, 06520-8107, USA
2. International Center for Theoretical Physics, Strada Costiera 11, 34151 Trieste, Italy
3. Laboratory of Computational Biology, National Heart, Lung and Blood Institute, National Institutes of Health, Bethesda, MD, 20852, USA
4. Université de Lyon, CNRS, Institut de Chimie de Lyon, École Normale Supérieure de Lyon, Lyon Cedex 07, France,
5. Dipartimento di Chimica Industriale ‘‘Toso Montanari’’, Alma Mater Studiorum, Università di Bologna, Bologna, Italia,
6. Department of Molecular Biophysics and Biochemistry, Yale University, New Haven, CT 06520, USA

[*]authors contributed equally to this work
[a] corresponding author
✝current address: Novartis Institutes of BioMedical Research, 181 Massachusetts Avenue, Cambridge, MA 02139

Additional details on Experimental NMR chemical shift and Random Coil Indices:

NMR and RCI values are reported in the Source Data file (Sheet:Figure 3a), provided as supporting information.

NMR shifts are also publicly available as part of the BRMB database under the accession code: 15741

Chemical shift values obtained for the $^{1}H^{N}$, $^{15}N^{H}$, $^{13}C^{\alpha}$, and $^{13}C'$ nuclei corresponding to each amino acid residue for the apoenzyme at 30ºC and 50ºC as well as the holoenzyme at 30ºC were submitted to the RCI server[4] generating a random coil index. RCI values thus calculated were used as a metric to quantify changes in protein flexibility upon temperature increase and effector binding.

Codes to reproduce the analyses, are provided within the git repository: https://github.com/Batista-Chemistry-Lab/heatIGPS.

The description of the repository's structure is provided at https://github.com/Batista-Chemistry-Lab/heatIGPS/README.md.

Supplementary Methods

Molecular Dynamics Simulations:
Molecular dynamics simulations of the APO (apo30, apo50), and PRFAR (holo30, holo50) bound structures of IGPS are based on the CHARMM36m[1,2] and the generalized CHARMM force fields[3] using the CHARMM-GUI.[4] For the production run MD simulations we employed the following pre-equilibration procedure: minimization of hydrogen atoms and explicit TIP3 water, solvation of protein-complex in a water box of approximately 20000 water molecules and Na+, Cl- ions to neutralization, and subsequent minimization of the solvent constraining the rest of the atoms at the crystal structure positions (40 ps). The optimized solvated complex was then slowly heated to 303 K (for 30˚C simulations) or 323 K (for 50˚C simulations) for a minimum of 60 ps. Subsequent MD simulations (100 ps) in the canonical NVT ensemble were performed using Langevin dynamics, with applied harmonic constrains to the protein and PRFAR heavy atoms, with force constants set to 1 kcal/molÅ^{2}. During this heating procedure different parts of the system were gradually unconstrained until all atoms were set freed. Unconstrained MD simulations were run for more than 4 ns, for total pre-equilibration simulation time of at least 5 ns. Finally. MD simulations were performed in the NPT ensemble at 303 or 323 K and 1 atm using the Langevin piston. The MD simulations were carried out for at least 1200 ns in for replicas for each state. All simulations were performed using periodic boundary conditions. Electrostatic interactions were treated with the Particle Mesh Ewald method[5] and van der Walls interactions were calculated using a switching distance of 10 Å and a cutoff of 12 Å. We used a multiple time-stepping algorithm[6,7] to evaluate bonded, short-range nonbonded, and long-range electrostatic interactions at every one, two, and four timesteps, respectively, using a timestep of integration set to 2 fs.

NMR Sample Preparation: HisF-IGPS

Protein Overexpression: Plasmids containing *E.coli* codon-optimized genes for HisH and HisF from *Thermotoga maritima* cloned into pET43.1b vectors were purchased from GenScript by a previous graduate student in the Loria group.[8] Plasmids encoded with the HisH sequence (containing a C-terminal histidine tag) and the HisF sequence were transformed into BL21(DE3) cells. Samples isotopically enriched in ^{2}D, ^{15}N, and ^{13}C were prepared by growing BL21(DE3) cells overexpressing the HisF subunit in 1 liter of deuterated M9 minimal media, with $^{15}NH_4Cl$ and ^{13}C-glucose (Cambridge Isotope Labs, MA) as the sole nitrogen and carbon source, respectively. Samples enriched in ^{2}D and ^{15}N were grown in deuterated M9 minimal media supplemented solely with $^{15}NH_4Cl$ as the nitrogen source. The HisH subunit was grown in

deuterated minimal media with naturally abundant nitrogen and carbon isotopes. The cultures were grown to an OD_{600} of 0.8-1.0, induced with 1mM IPTG (Isopropyl β-d-1-thiogalactopyranoside, Sigma-Aldrich), and shaken in an incubator for 14-16 hours at 30 °C.

Purification: Cells were harvested by centrifugation at 4000 rpm for 35 minutes. Pellets for both HisH and HisF were resuspended in lysis buffer (10 mM Tris hydrochloride, 10 mM CAPS, 300 mM NaCl, and 1 mM β-mercaptoethanol, pH 7.5) and co-lysed by ultrasonication. The homogenized suspension was then incubated at 333K for 30 minutes to remove undesired proteins since IGPS is thermally stable at this temperature. The cell lysate was spun down using an ultracentrifuge to remove cell debris and the supernatant containing the soluble proteins was separated. The supernatant was incubated over a bed of Ni-NTA agarose resin for 20 minutes equilibrated with the same lysis buffer described above. This supernatant-resin mixture was then added to a gravity column and washed with 5 column volumes of lysis buffer. The column was subsequently washed with 15 column volumes of wash buffer (10 mM Tris hydrochloride, 10 mM CAPS, 300 mM NaCl, 1 mM β-mercaptoethanol, 15 mM Imidazole, pH 9.5) and finally with 10 column volumes of elution buffer (10 mM Tris hydrochloride, 10 mM CAPS, 300 mM NaCl, 1 mM β-mercaptoethanol, and 15 mM Imidazole, pH 9.5) to elute IGPS. The eluent was then dialyzed against dialysis buffer overnight and concentrated further to be used for NMR experiments. The dialysis buffer used for NMR experiments contained 10 mM HEPES, 10 mM KCl, and 0.5 mM EDTA at a pH of 7.3.

Supplementary Figures and Tables

<table>
<tr><th>SideR (red)</th><th>SideL (blue)</th><th>Ambiguous (white)</th></tr>
<tr><td colspan="3">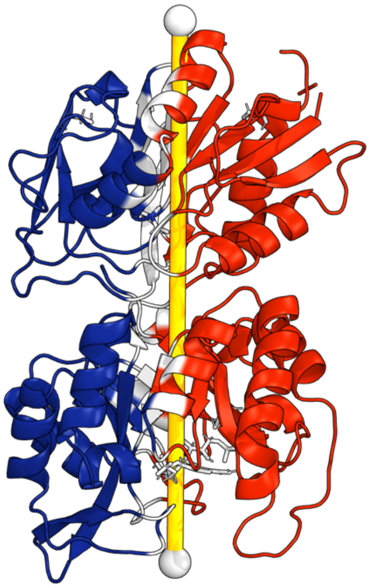</td></tr>
<tr>
<td>ARG:5, ILE:6, ILE:7, ALA:8, CYS:9, LEU:10, ASP:11, VAL:12, LYS:13, ASP:14, GLY:15, ARG:16, VAL:17, VAL:18, LYS:19, GLY:20, THR:21, ASN:22, PHE:23, GLU:24, ASN:25, LEU:26, ARG:27, ASP:28, SER:29, GLY:30, ASP:31, PRO:32, VAL:33, GLU:34, LEU:35, GLY:36, LYS:37, PHE:38, TYR:39, SER:40, GLU:41, ILE:42, GLY:43, ILE:44, ASP:45, GLU:46, LEU:47, VAL:48, PHE:49, LEU:50, ASP:51, ILE:52, THR:53, ALA:54, SER:55, VAL:56, GLU:57, LYS:58, ARG:59, LYS:60, THR:61, MET:62, LEU:63, GLU:64, LEU:65, VAL:66, GLU:67, LYS:68, VAL:69, ALA:70, GLU:71, GLN:72, ILE:73, ASP:74, ILE:75, PRO:76, PHE:77, THR:78, VAL:79, GLY:80, GLY:81, GLY:82, ILE:83, THR:88, ALA:89, GLU:91,</td>
<td>MET:1, LEU:2, ASP:85, PHE:86, ASN:103, THR:104, ALA:105, ALA:106, VAL:107, GLU:108, ASN:109, PRO:110, SER:111, LEU:112, ILE:113, THR:114, GLN:115, ILE:116, ALA:117, GLN:118, THR:119, PHE:120, GLY:121, SER:122, GLN:123, VAL:125, VAL:126, VAL:127, ALA:128, ILE:129, ASP:130, ALA:131, LYS:132, ARG:133, VAL:134, ASP:135, GLY:136, GLU:137, PHE:138, MET:139, VAL:140, PHE:141, THR:142, GLY:145, LYS:147, ASN:148, THR:149, GLY:150, ILE:151, LEU:152, LEU:153, ARG:154, ASP:155, TRP:156, VAL:157, VAL:158, GLU:159, VAL:160, GLU:161, LYS:162, ARG:163, GLY:164, ALA:165, GLY:166, GLU:167, ILE:168, LEU:169, LEU:170, SER:172, ASP:183, THR:184, GLU:185, MET:186, ILE:187, ARG:188, PHE:189, VAL:190, ARG:191, PRO:192, LEU:193, THR:194,</td>
<td>ALA:3, LYS:4, HIE:84, GLU:87, SER:90, LYS:99, SER:101, ILE:102, ALA:124, TYR:143, SER:144, LYS:146, THR:171, ILE:173, ASP:174, ARG:175, TYR:182, ILE:199, SER:201, HIE:209, LEU:211, ALA:220, ALA:221, LYS:242, HIE:244, ASN:247, VAL:248, GLY:262, PRO:263, GLY:264, GLY:303, ARG:313, LEU:314, ASP:318, ILE:320, ASP:321, ARG:324, GLY:335, VAL:336, GLY:339, GLY:375, TRP:376, ASN:377, GLU:378, VAL:379, ILE:380, PHE:381, LYS:382, TYR:391, PHE:392, LEU:406, GLY:407, ALA:419, ARG:421, GLY:423, LEU:426, GLY:427, PHE:428, PHE:430
(PRFAR is shown in sticks)</td>
</tr>
</table>

LEU:92, ILE:93, LEU:94, ARG:95, GLY:96, ALA:97, ASP:98, VAL:100, ASP:176, GLY:177, THR:178, LYS:179, SER:180, GLY:181, GLY:202, GLY:203, ALA:204, GLY:205, LYS:206, MET:207, GLU:208, PHE:210, LEU:222, ALA:223, ALA:224, SER:225, VAL:226, PHE:227, HIE:228, PHE:229, ARG:230, GLU:231, ILE:232, ASP:233, VAL:234, ARG:235, GLU:236, LEU:237, LYS:238, GLU:239, TYR:240, LEU:241, LYS:243, ARG:249, LEU:250, GLU:251, GLY:252, LEU:253, MET:254, ARG:255, ILE:256, GLY:257, ILE:258, ILE:259, SER:260, VAL:261, ASN:265, ILE:266, MET:267, ASN:268, LEU:269, TYR:270, ARG:271, GLY:272, VAL:273, LYS:274, ARG:275, ALA:276, SER:277, GLU:278, ASN:279, PHE:280, GLU:281, ASP:282, VAL:283, SER:284, ILE:285, GLU:286, LEU:287, VAL:288, GLU:289, SER:290, PRO:291, ARG:292, ASN:293, ASP:294, LEU:295, TYR:296, ASP:297, LEU:298, LEU:299, PHE:300, ILE:301, PRO:302, ASN:317, LEU:319, PHE:322, VAL:323, LYS:325, HID:326, VAL:327, GLU:328, ASP:329, GLU:330, ARG:331, TYR:332, VAL:333, VAL:334, ASP:383, THR:384, PHE:385, PRO:386, ASN:387, GLY:388, TYR:389, TYR:390, ARG:424, ILE:425, HIE:431, PRO:432, GLU:433, LYS:434, SER:435, SER:436, LYS:437, ILE:438, GLY:439, ARG:440, LYS:441, LEU:442, LEU:443, GLU:444, LYS:445, VAL:446, ILE:447, GLU:448, CYS:449, SER:450, LEU:451, SER:452, ARG:453, ARG:454	THR:195, LEU:196, PRO:197, ILE:198, ALA:200, GLU:212, ALA:213, PHE:214, LEU:215, ALA:216, GLY:217, ALA:218, ASP:219, GLY:245, VAL:246, VAL:304, GLY:305, HIE:306, PHE:307, GLY:308, GLU:309, GLY:310, MET:311, ARG:312, ARG:315, GLU:316, CYS:337, LEU:338, MET:340, GLN:341, LEU:342, LEU:343, PHE:344, GLU:345, GLU:346, SER:347, GLU:348, GLU:349, ALA:350, PRO:351, GLY:352, VAL:353, LYS:354, GLY:355, LEU:356, SER:357, LEU:358, ILE:359, GLU:360, GLY:361, ASN:362, VAL:363, VAL:364, LYS:365, LEU:366, ARG:367, SER:368, ARG:369, ARG:370, LEU:371, PRO:372, HID:373, MET:374, VAL:393, HID:394, THR:395, TYR:396, ARG:397, ALA:398, VAL:399, CYS:400, GLU:401, GLU:402, GLU:403, HIE:404, VAL:405, THR:408, THR:409, GLU:410, TYR:411, ASP:412, GLY:413, GLU:414, ILE:415, PHE:416, PRO:417, SER:418, VAL:420, LYS:422, GLN:429	

Supplementary Table 1. List of residues catalogued as SideL, SideR and Ambiguous. The partition is based on the position of residues relative to the plane dividing the protein structure in two halves and lying on the yellow axis shown in the figure. Residues that lie on the right of the axis, at a perpendicular distance greater of equal of 3 Å from the axis are defined as belonging to side R. Residues that lie on the left of the axis, at a perpendicular distance greater of equal of 1 Å from the axis are defined as belonging to side L. All other residues are defined as ambiguous.

To improve the overall statistics, we performed four 1μs simulation replicas of (also) holo50, providing enough statistics to analyze the unbinding dynamics. We computed the relative distance between the center of mass of PRFAR and the effector site (i.e. protein residues located in a sphere radius of 6 Å from PRFAR) along the trajectories, and we reported the results in Supplementary Fig. 1. At 50˚C, the distance between the two centers of mass varies greatly across the trajectories, suggesting that PRFAR explores different positions, in contrast to what occurs at 30˚C (see Supplementary Fig. 1A). PRFAR dynamics in holo50 features molecular interactions with different residues, holding the effector bound to the protein while not necessarily in its binding pocket. Only very rarely, the effector features broken HBs (min d > 2 Å) with the HisF protein (see Supplementary Fig. 1A bottom). As reported in the new Supplementary Fig. 1C, we found that the number of hydrogen bonds per frame in the holo50 simulations is comparable to that of apo30. The number of residues involved in hydrogen bonds, however, increases at elevated temperature conditions. The results thus indicated increased mobility of PRFAR at higher temperature, leading to significant displacements in the pocket.

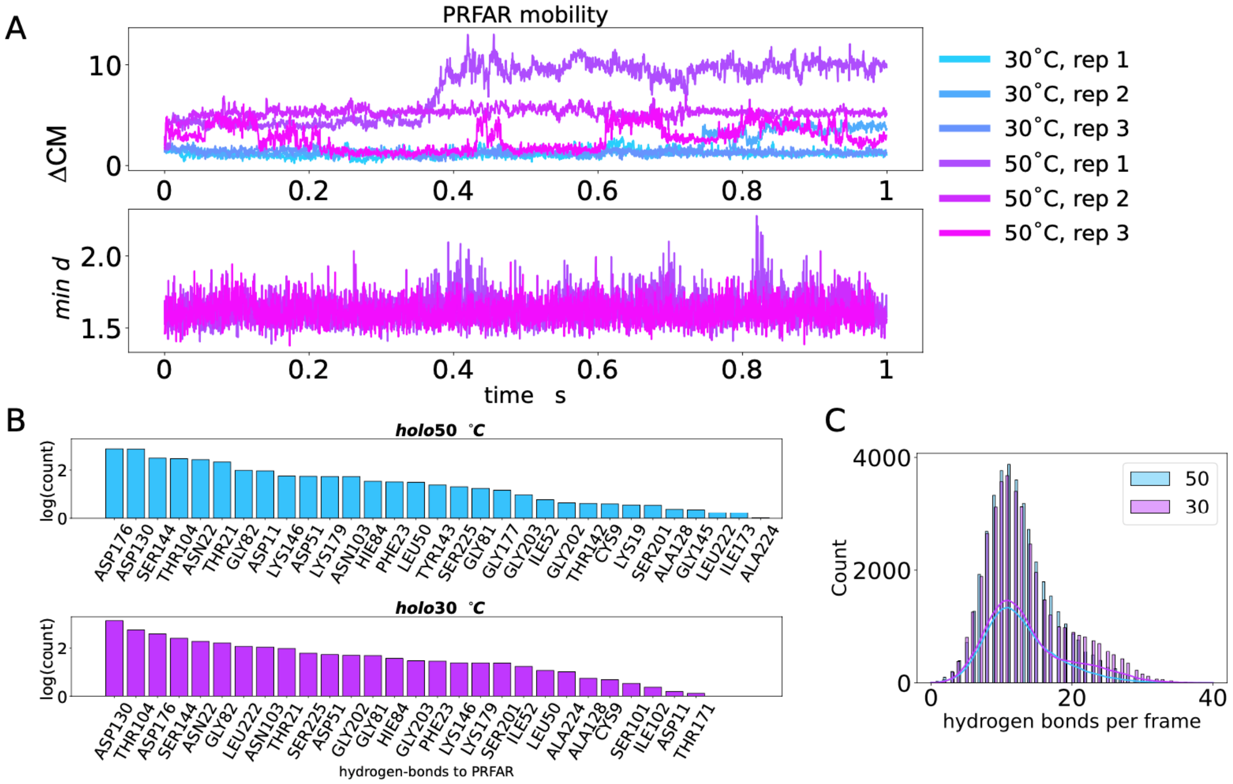

Supplementary Fig. 1. PRFAR mobility. (A) Relative distance between the center of mass of PRFAR and the effector site (residues located in a sphere radius of 6 Å from PRFAR), over the course of three additional 1μs long holo30 and *holo50* trajectories (ΔCM), and minimum distance (min d) between any atom of PRFAR and any atom of the protein at each frame of the trajectories. It is apparent that at 50 ˚C the distance between the two centers of mass varies greatly across the trajectories, suggesting that PRFAR explores different positions throughout the trajectories. (B) PRFAR dynamics in holo50 features molecular interactions with different residues, holding the effector bound to the protein while not necessary in its binding pocket. (C) Kernel density estimate of hydrogen bond counts per frame.

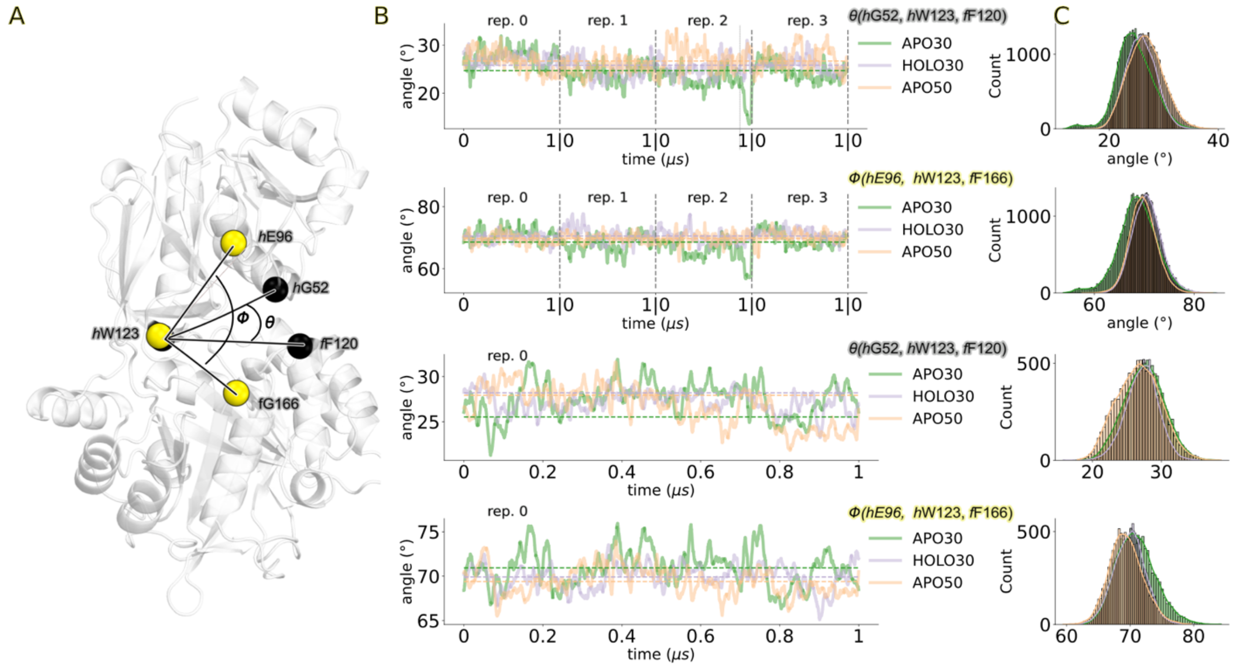

Supplementary Fig. 2. **Hinge-motion** (A) Instantaneous representation of the breathing motion angles (between Cα atoms of *f*F120, *h*W123, *h*G52 and *h*E96, *h*W123, *f*G166) during the first frame of the apo30 simulation. (B) Breathing motion angle evolution during the four independent 1μs replicas of MD simulation (top two rows) in APO30 (green), APO50 (orange), and HOLO30 (purple) with moving average with a time window of 100 frames=10ns. The mean values of each distribution are shown as dotted lines with matching colors. (C) Kernel density estimate of the distribution of breathing motion angle in each trajectory. The bottom rows in panel B and C show a closeup of the angle distributions computed only for a representative MD simulation (replica 0).

When a single replica is considered one can only appreciate that the holo distribution appears narrower than the other two, suggesting that PRFAR reduces probability of larger angles. The effect is more pronounced when all replicas are considered, where irrespective of the angle considered, the APO trajectories sample significantly more “close” conformation than what observed in presence of either activator. Moreover, temperature and PRFAR distributions show substantial overlap, which supports the hypothesis that the temperature mimics PRFAR.

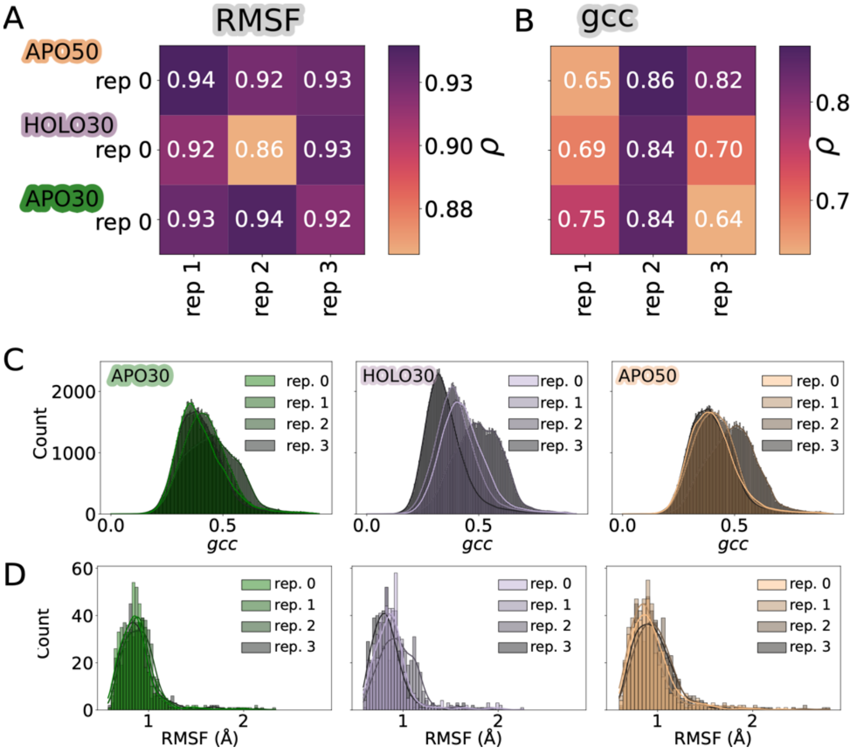

Supplementary Fig. 3. Reproducibility and reliability of sampled conformations: **correlation between replica 0 (reported in main text) and subsequent independent replicas of *apo30, holo30* and *apo50* states.** A) Spearman correlation coefficient calculated between the Cα RMSF distributions of the apo30, apo50, holo30 (rep. 0) and the corresponding distributions extracted from three additional replicas (rep. 1, rep. 2, rep 3.). RMSF distributions are computed upon alignment of each trajectory to the average Cα positions. **B**) Spearman correlation coefficient calculated between mutual information based generalized correlation coefficients (gcc) distributions of the apo30, apo50, holo30 (rep. 0) and the corresponding distributions extracted from three additional replicas (rep. 1, rep. 2, rep 3.). Gcc values are the upper triangular entries of the mutual information-based correlation matrices computed for Cα displacements of each simulation. C) Kernel density estimate of the gcc distributions of each trajectory. D) Kernel density estimate of the RMSF distributions of each trajectory. Both gcc and RMSF distributions show substantial overlap across different replicas.

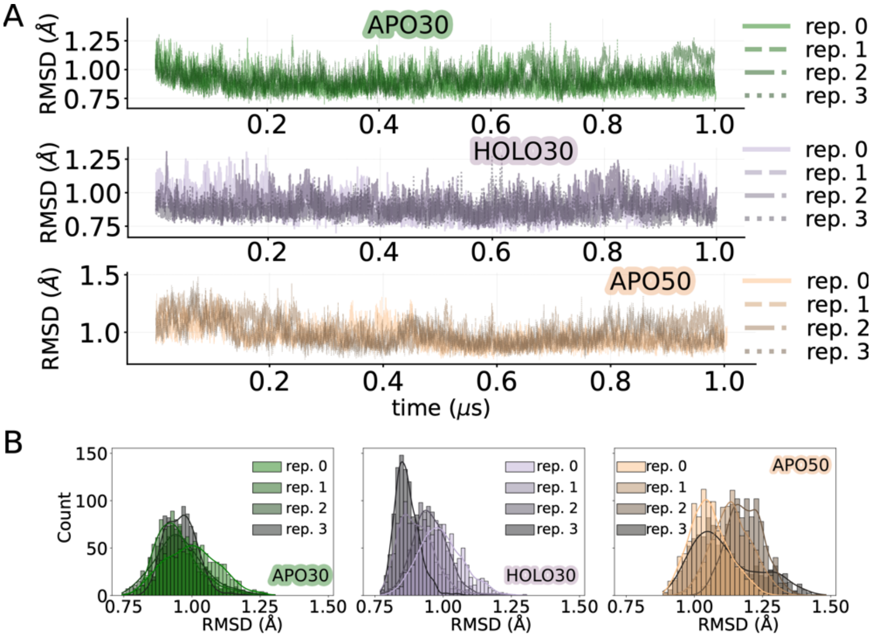

Supplementary Fig. 4. Reproducibility and reliability of sampled conformations: RMSD profiles of Cα trajectories of *apo30, holo30* and *apo50* states computed for four independent replicas of each state, using the average as reference structure. RMSD profiles are computed upon alignment of each trajectory to the average Cα positions of each residue. A) RMSD curves for four replicas of apo30, apo50 and holo30 simulations. B) Kernel density distributions for the RMSD curves of the four replicas of apo30, apo50 and holo30 states.

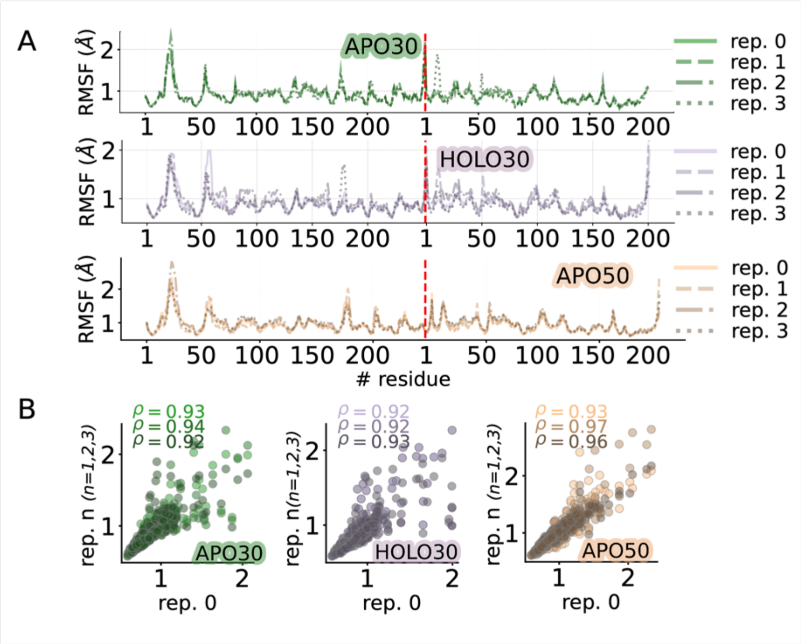

Supplementary Fig. 5. Reproducibility and reliability of sampled conformations: RMSF profiles of Cα trajectories of *apo30, holo30* and *apo50* states computed for four independent replicas of each state. RMSF profiles are computed upon alignment of each trajectory to the average Cα positions of each residue.

A) RMSF curves for four replicas of apo30, apo50 and holo30 simulations. The red dotted lines indicate the separation between HisF and HisH B) Scatter plots showing the correlation between RMSF distribution relative to replica 0 and replicas 1,2,3 of apo30, apo50 and holo30 states. The computed spearman correlation coefficients are reported with corresponding colors. The computed root mean square fluctuations relative to replicas 1,2,3 are highly correlated to that of replica 0, with spearman correlation coefficients all greater than 0.9.

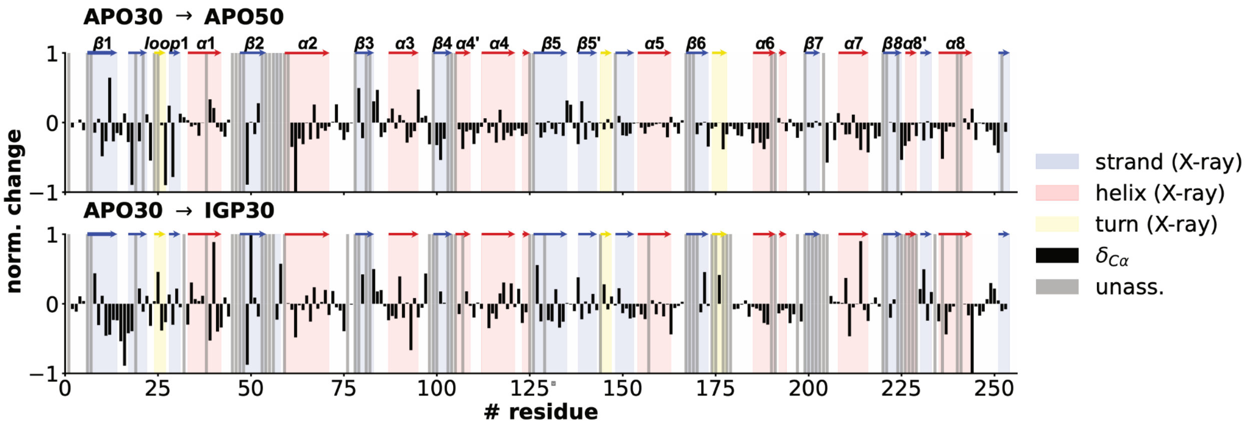

Supplementary Fig. 6. Experimental NMR difference Ca assignments. (Top) apo30 to apo50 difference in Cα chemical shifts. (Bottom) apo30 to IGP bound (at 30 degrees) difference in Cα chemical shifts. NMR shifts for the PRFAR-bound system cannot be collected experimentally due to exchange broadening of nearly half of the assigned resonances. Hence, we characterized the holo system using a Imidazole glycerol phosphate (the reaction product and also allosteric effector). Residues that cannot be assigned unambiguously or are exchanged broadened are shown as gray bars.

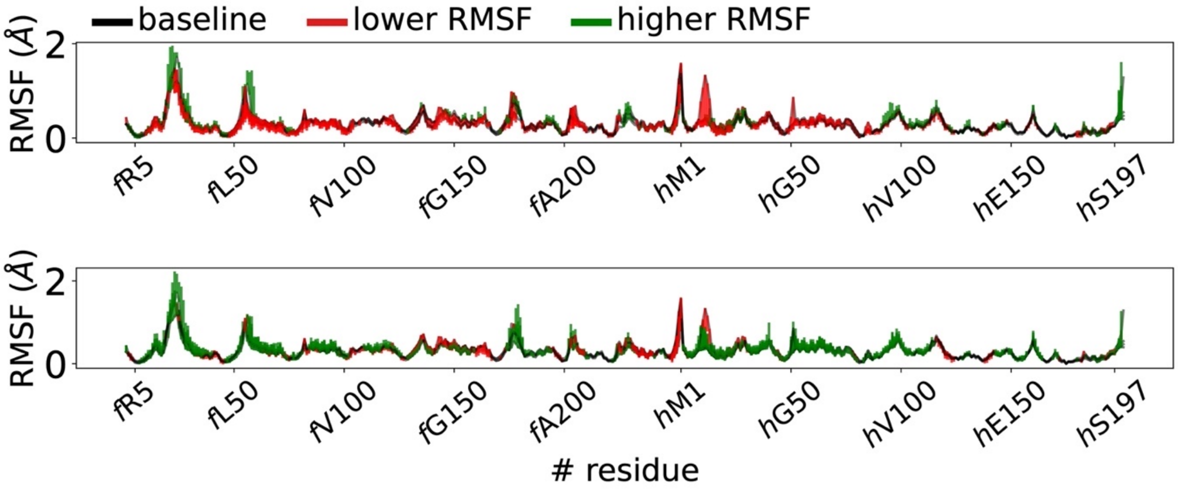

Supplementary Fig. 7. **RMSF analysis.** RMSF of apo30 (black lines, top and bottom) compared to holo30 (top) and apo50 (bottom) with green upward bars if the RMSF is bigger and red downward bars if the RMSF is lower. The curves are obtained by overlay of the RMSF curves relative to each of the four independent replicas and their differences are shown.

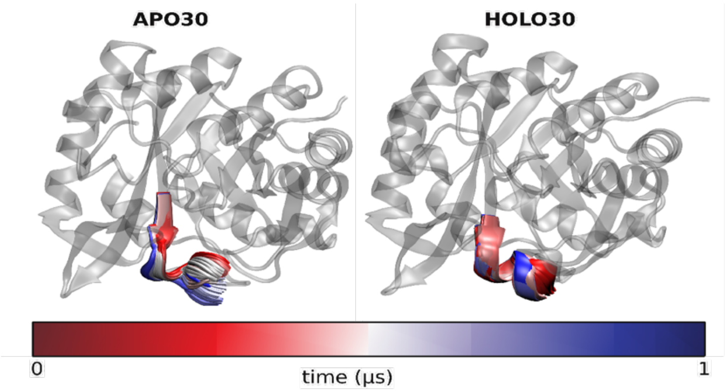

Supplementary Fig. 8. Formation of *fα*6' along the trajectory. Time representation constructed using one frame every 10 ns of a representative simulation (replica 0) showing the formation of *fα*6' in the PRFAR-bound (*holo30*) simulation, as opposed to the absence of ordered secondary structure in the absence of the effector (apo30).

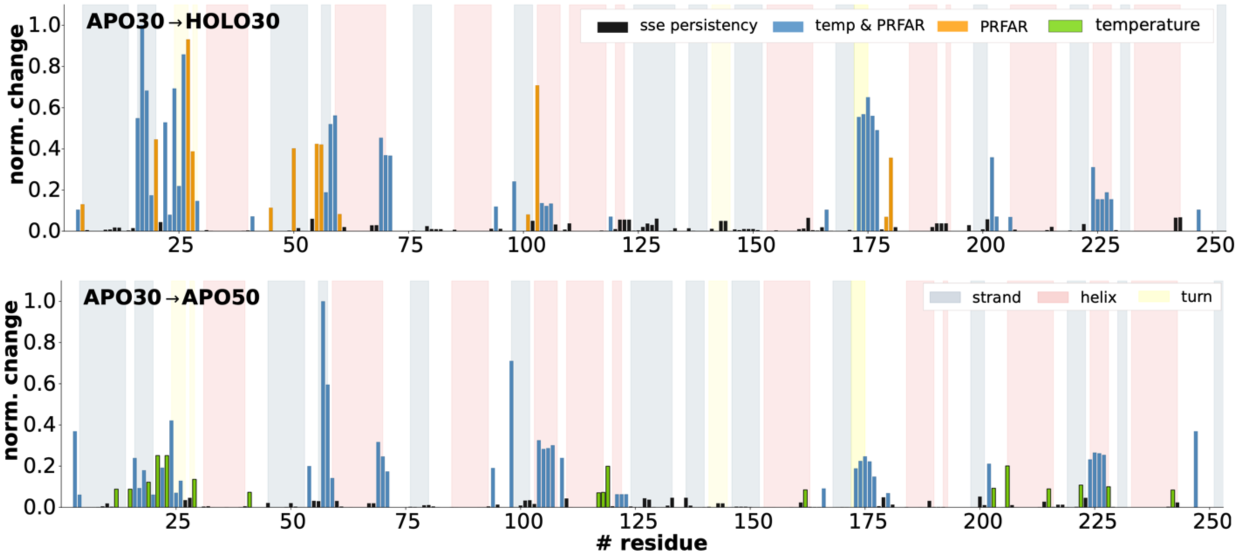

Supplementary Fig. 9. Effect of PRFAR and temperature-dependent activation of IGPS. (Top) Change in secondary structure element (SSE) persistency for each residue induced by PRFAR. SSE persistency values are computed as the percentage of frames in which each residue retains the secondary structure as assigned in the 1GPW crystal structure. (bottom) Analogous to (top) but depicting the SSE changes occurring from *apo30* to *apo50*.

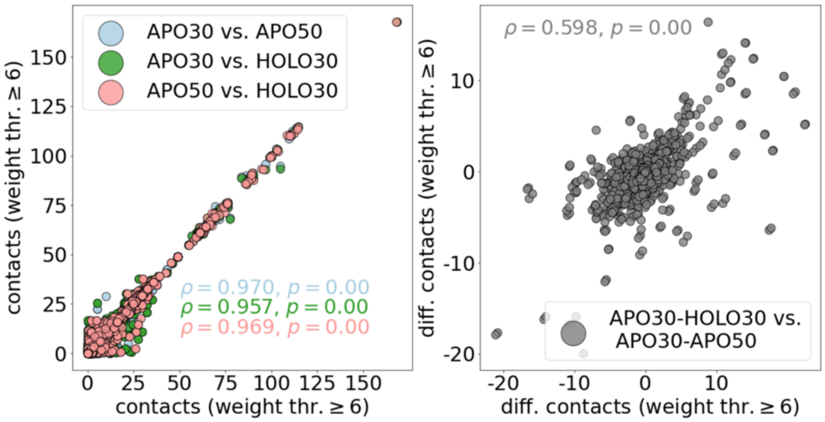

Supplementary Fig. 10. (Left) Correlation plot between the weight of selected edges in the DCPN between *apo30* and *holo30, apo30* and *apo50, apo50* and *holo30*. The selected edges include those with weight greater or equal to 5 in either of the three sets. (Right) Scatterplot showing the correlation between the apo30-

holo30 and apo30-apo50 differences from a random network, computed considering the same selection.

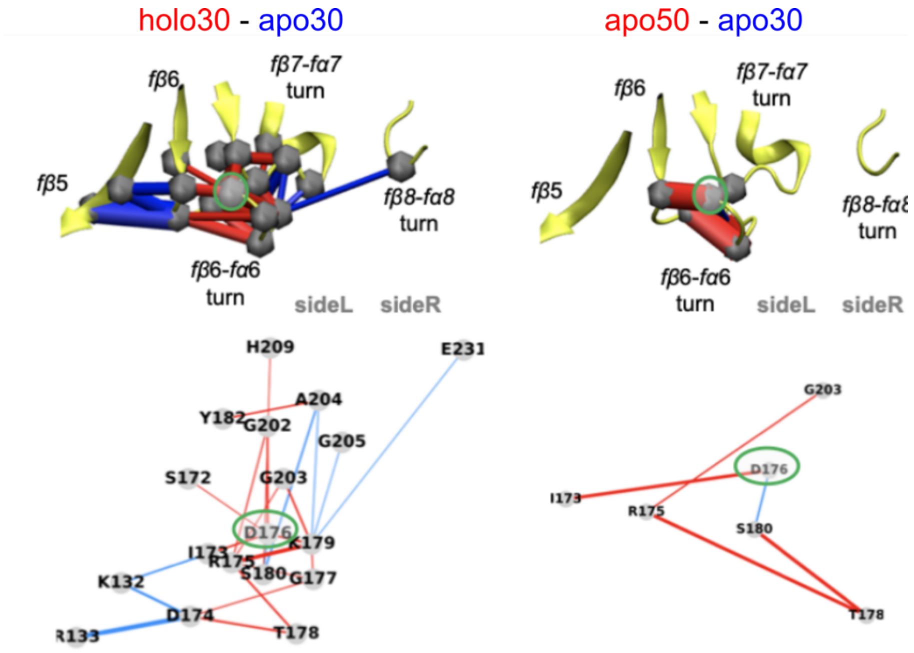

Supplementary Fig. 11 Induced perturbations. Induced perturbations for *f*D176 for the apo30/holo30 (left panel) and apo30/apo50 (right panel) perturbation networks. Blue and red edges represent a bigger number of contacts in the systems labeled with blue and red text, respectively. Edge widths are proportional to the number of contact changes.

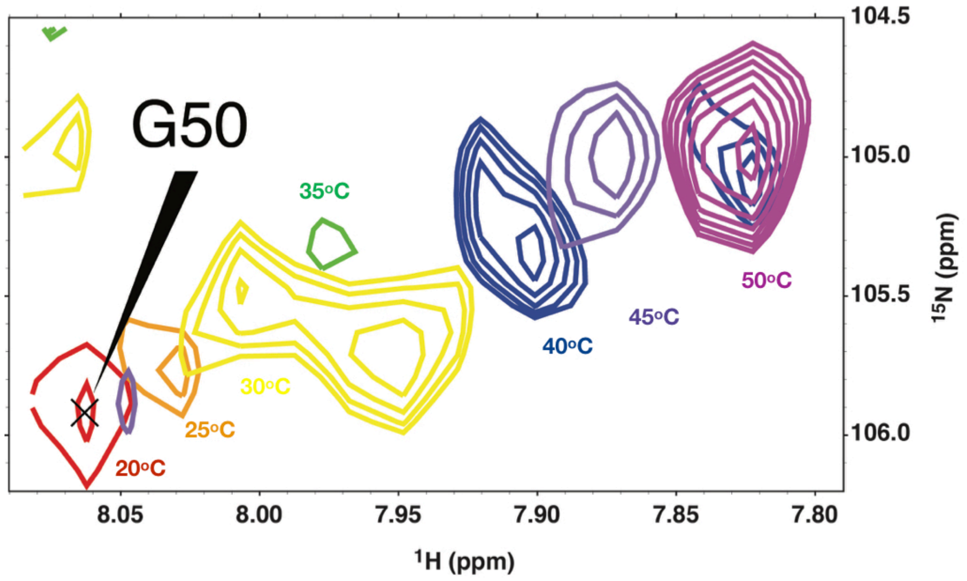

Supplementary Fig. 12. Temperature dependence of G50. Section of a two-dimensional TROSY spectrum of 15N,2H HisH in complex with 2H-labeled HisF showing active site residue G50. As the temperature increases, the G50 resonances is severely broadened at 35ºC (green) and narrows at higher temperatures as it moves into the fast timescale NMR regime.

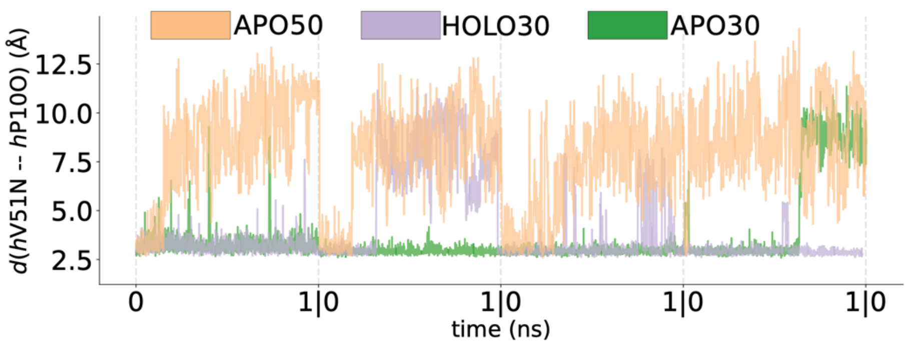

Supplementary Fig. 13. Distance profile of V51N--P10O measured across four 1µs replicas of *apo30, holo30* and *apo50* simulations. The mean value of each distribution is shown as a solid line of corresponding color. The dotted lines represent the mean + standard deviations of each distribution.

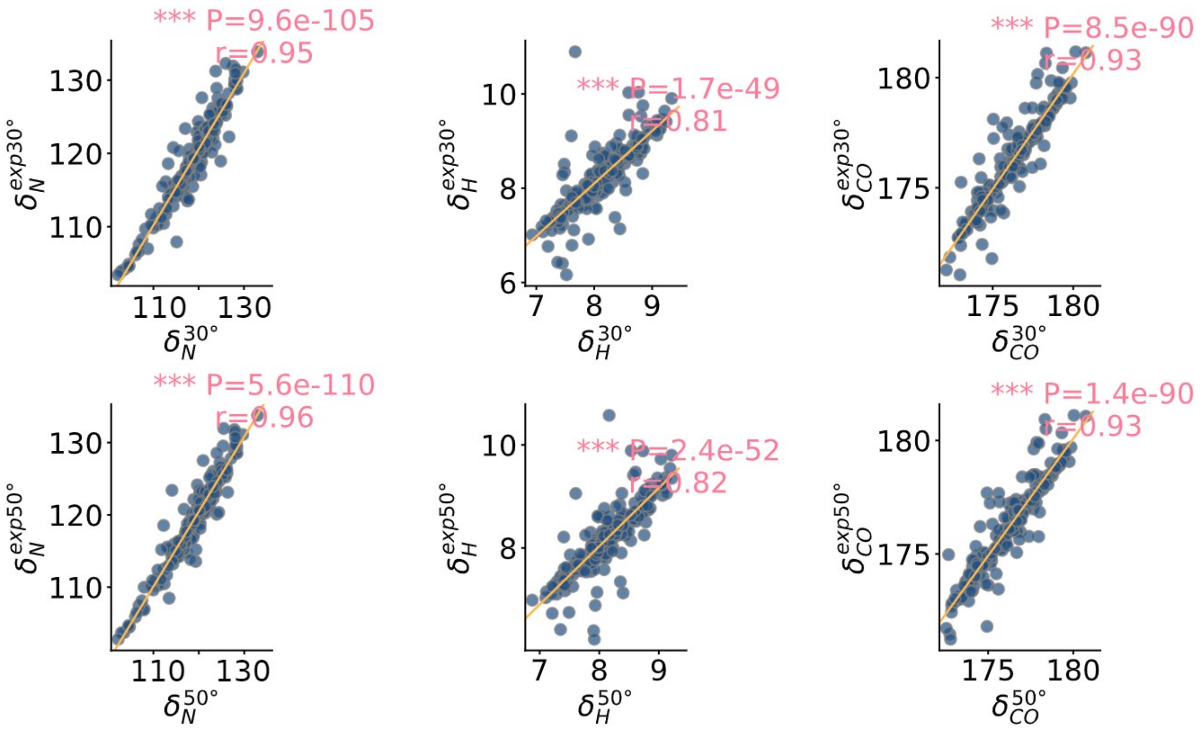

Supplementary Fig. 14. Correlations between ShiftX2 simulated chemical shifts computed for a representative 1µs simulation (replica 0) and experimental chemical shifts for N, H, and C' at 30°C and 50°C. The Pearson correlation coefficients and corresponding two-sided P-values are shown.

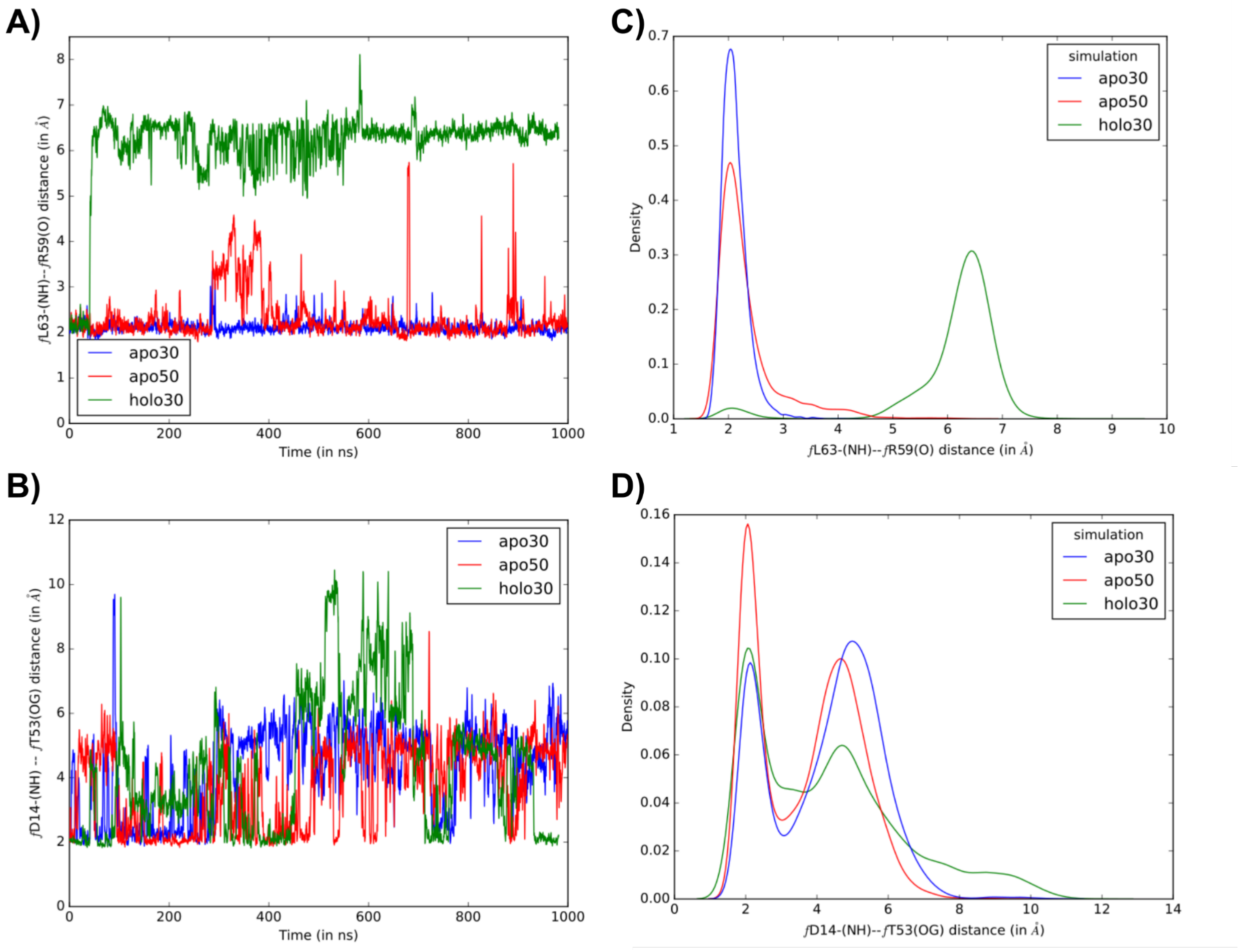

Supplementary Fig. 15. Hydrogen bond distance (A) along the 1μs representative simulation of apo30 (blue) and apo50 (red) between residues *f*L63-*f*R59 (A) and *f*D14-*f*T53 (B). Kernel density estimate of the length of the respective same hydrogen bonds (C and D).

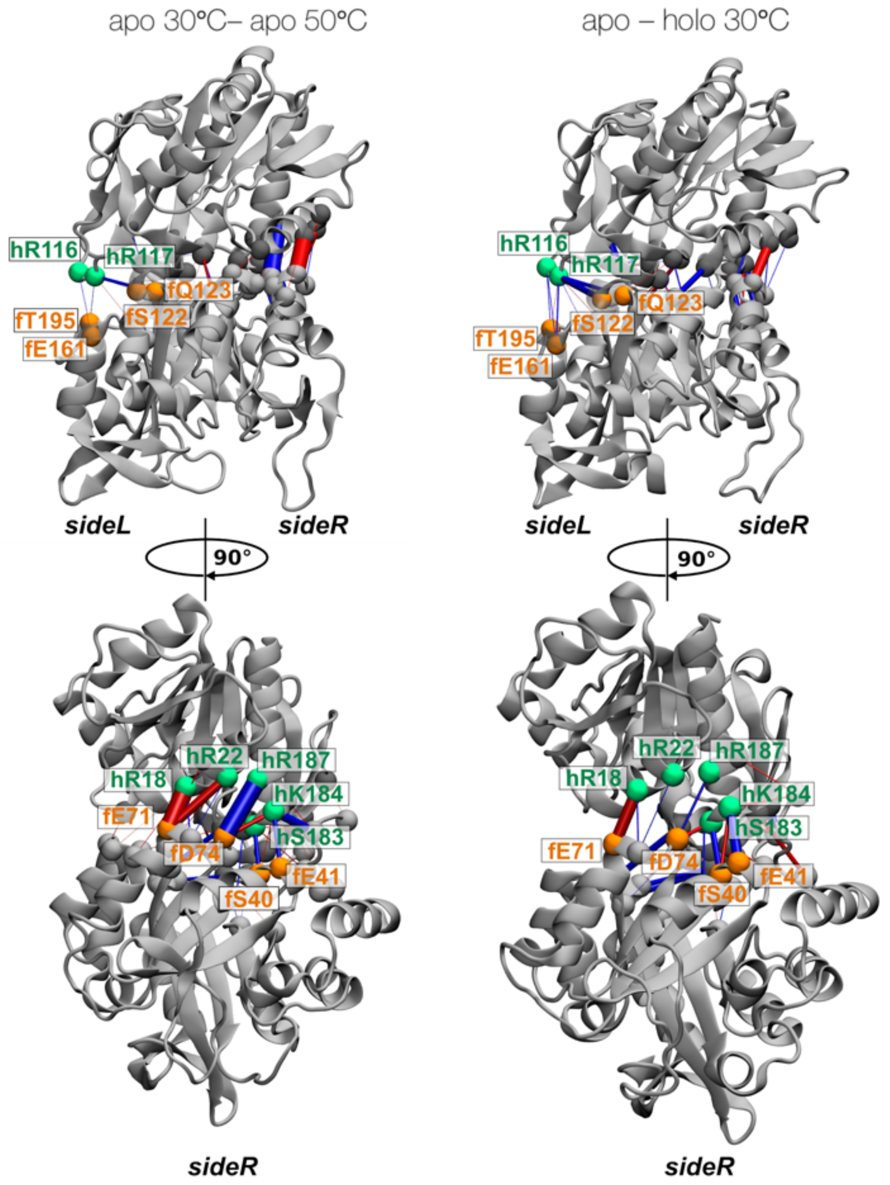

Supplementary Fig. 16. **Hydrogen bonding at the hisF-hisH interface**. The upper panel shows the HB network on sideL, bottom panel represents the HB networks at sideR. Residues that form strong HB in HisF and in HisH are represented as orange spheres, and green spheres, respectively. A blue cylinder indicates that the HBs were more persistent in the apo30 simulation, whereas a red cylinder indicates more HBs for the apo50 simulation, or the holo30 simulation. The hydrogen bond (HB) analysis was performed by using PyHVis3D,[9] a python-based package to calculate pairwise HBs between all donors and acceptors of all frames of the simulation trajectory.[8] The distance cutoff between acceptor and donor is 3.5 Å and the angle cutoff hydrogen–donor–acceptor is 30º. The algorithm calculates an N*N matrix (N = the number of donor/acceptor atoms in the protein), and each matrix element represents the average presence of a HB between two atoms over the simulation time. Overall, the pattern of hydrogen bonding once again evidences the strong structural parallelism between temperature increase from 30ºC to 50ºC and PRFAR binding at 30 ºC.

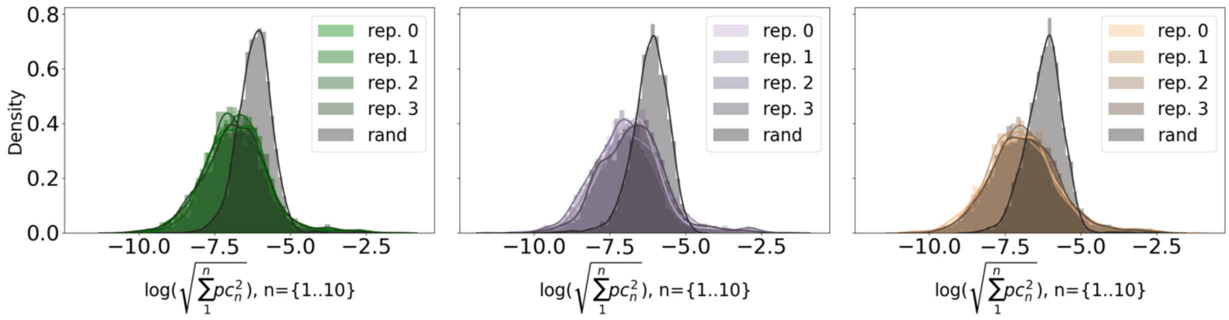

Supplementary Fig. 17. Kernel density distributions of selected principal components computed for four independent replicas of MD simulations of apo30 (green), holo30 (purple) and apo50 (orange) states. The distributions are calculated using the ten principal components corresponding to the largest ten eigenvalues of the covariance matrix computed for each trajectory. The distributions are compared to that obtained choosing 10 random eigenvectors from the diagonalized covariance matrix of replica 0. In all cases, we observe substantial overlap between the eigenvector's distributions of each different replica.

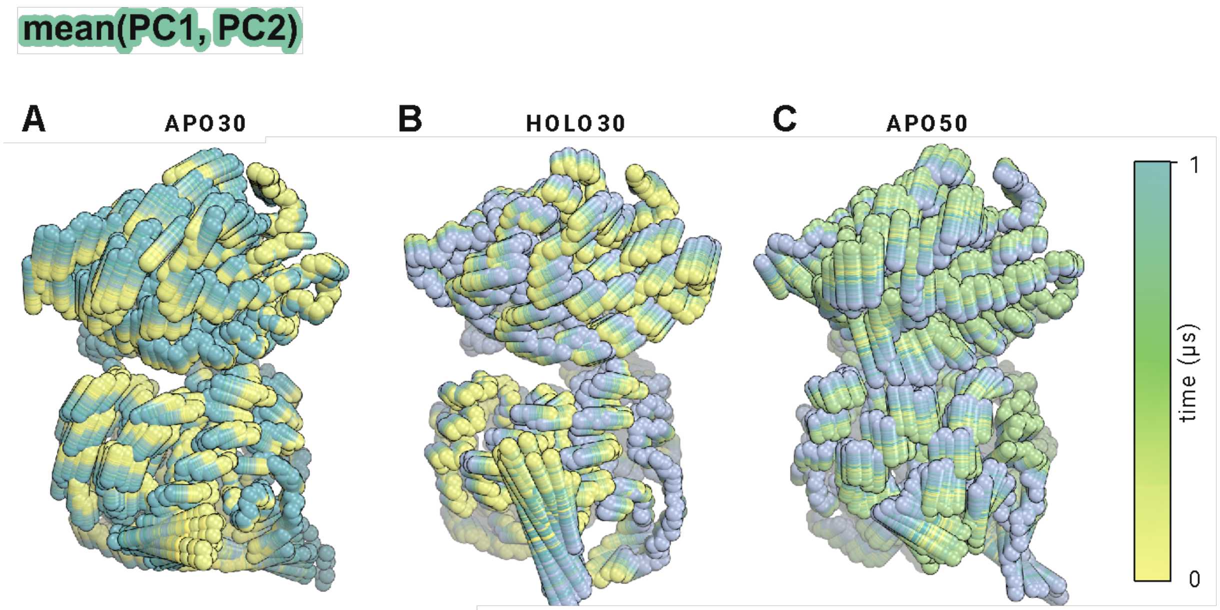

Supplementary Fig. 18. Essential motions of *apo30* (A), *holo30* (B), and *apo50* (C) across replica 0 of each state. The trajectories were obtained by projecting each trajectory in the space described by *mean(PC1, PC2)*. The overlay of 100 structures (one every 10 ns) reproduces the motion described by the principal components over 1 µs time of each trajectory.

Supplementary References

1. Huang, M., Giese, T. J., Lee, T.-S. & York, D. M. Improvement of DNA and RNA Sugar Pucker Profiles from Semiempirical Quantum Methods. *J. Chem. Theory Comput.* **10**, 1538–1545 (2014).
2. Huang, J. *et al.* CHARMM36m: an improved force field for folded and intrinsically disordered proteins. *Nat. Methods* **14**, 71–73 (2017).
3. Vanommeslaeghe, K. *et al.* CHARMM general force field: A force field for drug-like molecules compatible with the CHARMM all-atom additive biological force fields. *J. Comput. Chem.* **31**, 671–690 (2010).
4. Lee, J. *et al.* CHARMM-GUI Input Generator for NAMD, GROMACS, AMBER, OpenMM, and CHARMM/OpenMM Simulations Using the CHARMM36 Additive Force Field. *J. Chem. Theory Comput.* **12**, 405–413 (2016).
5. Darden T, York D, Pedersen, Particle mesh Ewald: an N-log(N) method for Ewald sums in large systems. J Chem Phys **89**,10089–10092 (1993).
6. Grubmuller H, Heller H, Windemuth H, Schulten K, Generalized Verlet algorithm for efficient molecular dynamics simulations with long-range interactions. Mol Sim **6**,121–142. (1991).
7. Schlick T, et al., Algorithmic challenges in computational molecular biophysics. J Comput Phys **151**, 9–48 (1999).
8. Lipchock, J.M. & Loria, J.P. Nanometer propagation of millisecond motions in V-type allostery. *Structure* **18**, 1596-607 (2010).
9. Knapp, B. *et al.* pyHVis3D: visualizing molecular simulation deduced H-bond networks in 3D: application to T-cell receptor interactions. *Bioinformatics* **34**, 1941–1943 (2018).
